# Optimal Sca-1-based procedure for purifying mouse adipose-derived mesenchymal stem cells with enhanced proliferative and differentiation potential

**DOI:** 10.3389/fcell.2025.1566670

**Published:** 2025-05-16

**Authors:** Xingyu Tao, Jialian Wang, Yuan Yan, Peifeng Cheng, Bin Liu, Huimin Du, Bailin Niu

**Affiliations:** ^1^ Department of Intensive Care Medicine, Chongqing Emergency Medical Center, Chongqing University Central Hospital, Chongqing Key Laboratory of Emergency Medicine, School of Medicine, Chongqing University, Chongqing, China; ^2^ Department of Oncology, The First Affiliated Hospital of Chongqing Medical University, Chongqing, China; ^3^ Department of Surgery, Brigham and Women’s Hospital and Harvard Medical School, Boston, MA, United States

**Keywords:** adipose-derived mesenchymal stem cells, Sca-1, vascular stem cells, heterogeneity, purification, magnetic activated cell sorting

## Abstract

**Introduction:**

Adipose-derived mesenchymal stem cells (ADSCs) are promising candidates for mesenchymal stem cell (MSC) therapy due to their ease of isolation from the stromal vascular fraction (SVF) of adipose tissue. However, traditional isolation methods often result in mouse ADSCs with low purity and significant heterogeneity contributing to inconsistencies in results from preclinical and clinical studies. This is partly attributed to the lack of consensus on their surface markers.

**Methods:**

This study compared three purification methods for isolating mouse ADSCs based on Sca-1 positivity—direct adherence (ADSC-A), magnetic cell sorting followed by adherence (ADSC-M), and adherence to the third generation followed by magnetic cell sorting (ADSC-AM). Third-generation ADSCs were evaluated for proliferative activity, differentiation potential, and functional enrichment using proliferation assays, trilineage differentiation assays, and RNA sequencing. Flow cytometry was employed to assess Sca-1 positivity and the expression of positive (CD44, CD90, CD29) and negative markers (CD31, CD45) in the fourth-generation ADSCs.

**Results:**

Among the three methods, ADSC-AM exhibited superior properties, including uniform morphology, enhanced proliferation, and over 95% expression of Sca-1 and CD29. While all methods supported trilineage differentiation, ADSC-AM demonstrated enhanced adipogenesis. Furthermore, RNA sequencing and pathway enrichment analysis revealed that ADSC-AM possessed unique potential in angiogenesis and immune regulation.

**Discussion:**

These findings suggest that the ADSC-AM method offers a simple and reproducible approach for obtaining high-purity mouse ADSCs with better functional properties and provide a fundamental reference for understanding mouse ADSCs surface marker profiles.

## 1 Introduction

Adipose-derived mesenchymal stem cells (ADSCs) were first isolated in 2001 from the stromal vascular fraction (SVF) of adipose tissue (Zuk et al., 2001). The SVF is a heterogeneous mixture of cells obtained from adipose tissue after removing mature adipocytes, connective tissue, blood, and other impurities. This fraction contains ADSCs (CD29+ CD90+ CD31− CD45−), along with endothelial progenitor cells (CD34+ CD31+ CD146+ CD45−), pericytes (CD146+ CD44+ CD29+ CD34− CD45−), preadipocytes (CD34+ CD45− CD31− CD146−), endothelial cells (CD31+ CD34−), smooth muscle cells (SMA+), lymphocytes (CD4+ CD8+), macrophages (CD45+ CD14+ CD34+ CD206+), and other cell types (Gimble et al., 2011; Guo et al., 2016; SundarRaj et al., 2015). In 2013, the International Society for Cellular Therapy (ISCT) and the International Federation for Adipose Therapeutics and Science (IFATS) issued a revised joint statement defining surface markers for ADSCs. The recommended primary stable positive markers include CD13, CD29, CD44, CD73, CD90, CD105 (expressed in >80% of ADSCs). CD34 was identified as a primary unstable positive marker, as its expression levels are variable. Primary negative markers include CD31, CD45, CD235a (<2%). Secondary positive markers were identified as CD10, CD26, CD36, CD49d, CD49e, while secondary low or negative markers include CD3, CD11b, CD49f, CD106, PODXL (Bourin et al., 2013). It is important to note CD34 was identified as a primary unstable positive marker due to its variable expression levels during *in vitro* culture of ADSCs. However, in the context of freshly isolated stromal vascular fraction (SVF), CD34 remains a useful marker for identifying specific subpopulations, such as endothelial progenitor cells and preadipocytes. Therefore, its classification as “unstable” pertains to its transient expression during *in vitro* expansion, not its role in initial SVF characterization. Besides, these criteria primarily are primarily applicable to human-derived ADSCs. Mice are the most commonly used experimental animals in biomedicine due to their simplicity and high degree of genetic and physiological homology with humans. However, a surface-marker consensus for mouse-derived ADSCs remains under development. In current preclinical studies, the heterogeneity of mouse ADSCs is often overlooked, despite its critical impact on research outcomes has not been recognized as a critical factor influencing research outcomes. Consequently, the application of mouse ADSCs to *in vivo* and *in vitro* models is often carried out in a generalized cell population form, resulting in inconsistent and unpredictable therapeutic outcomes in both clinical trials and studies (Chen et al., 2024). This variability has hindered the further development of ADSC-based clinical therapies.

The stem cell antigen-1 (Sca-1), or Ly-6A/E, is a glycosylphosphatidylinositol-anchored cell surface protein (GPI-AP) that was initially identified as a marker for murine hematopoietic cells. Sca-1 is now recognized as a marker expressed in various tissues, including skeletal muscle, myocardium, liver, and peripheral blood, where it is linked to self-renewal and pluripotency, making it widely accepted to enrich stem cells in a variety of tissues as a marker (Holmes and Stanford, 2007). Recent studies, such as those by Juan Tang et al., have shown that Sca-1+ cells contribute to smooth muscle regeneration in vascular walls after injury, with increased proliferative and vascular repair capacities. Lineage tracing studies revealed that Sca-1+ PDGFRα+ vascular stem cells (VSCs) in the epidermal stroma contribute to smooth muscle formation during vascular repair (Tang et al., 2020). Additional research indicates that Sca-1+ endothelial cells serve as a reserve population with enhanced proliferative and angiogenic potential (Tang et al., 2021). Although some researchers have considered the Sca-1+ cell population and MSCs in the blood vessels as two populations of vascular progenitor cells that jointly play a role in vascular remodeling (Kokkinopoulos et al., 2017), their overlap in localization and function suggests that Sca-1+ cells are a subset of MSCs with robust proliferation and differentiation abilities. This was partially confirmed in another study where Mižikova et al. used fluorescent RNA *in situ* hybridization to successfully target Sca-1+ pulmonary MSCs in the developing perivascular region of the lung (Mižíková et al., 2022), suggesting that perivascular Sca-1+ VSCs and Sca-1+ ADSCs likely overlap in localization and function within adipose tissue.

The traditional method for mouse ADSC isolation involves anatomical separation of adipose tissue followed by collagenase digestion, centrifugation, and adherence culture to remove non-adherent cells, which is adopted widely in many studies currently (Zuk et al., 2002; Andreeva et al., 2017; Li et al., 2023). However, this process often results in heterogeneous mouse ADSC population with various unidentified subtypes. The inability to fully eliminate endothelial, hematopoietic, and fibroblast-like cells makes it difficult to ensure mouse ADSC purity. The phenotype of mouse ADSCs is not clearly defined, and their characteristics can vary depending on tissue source, extraction method, and culture conditions. This heterogeneity complicates preclinical studies and reduces the reproducibility of experimental outcomes (Nicodemou and Danisovic, 2017). Therefore, selecting appropriate antigen markers is crucial for purifying mouse ADSCs and identifying specific subsets. Sca-1, is unlikely to be a universal stem/progenitor cell marker, but has proven to be an effective marker for enriching stem cell populations across various tissues (Holmes and Stanford, 2007; Hu et al., 2023). In the case of purifying mouse ADSCs, Sca-1 labeling offers several advantages, including enhanced proliferation, self-renewal potential, and long-term stability during cell culture (Tokunaga et al., 2014; Bonyadi et al., 2003), which is beneficial for batch expansion. Sca-1+ ADSCs also demonstrate superior trilineage differentiation potential (James et al., 2017; Staszkiewicz et al., 2008; Valorani et al., 2010) and support for immune and hematopoietic systems (Kayaba et al., 2018; Jatho et al., 2022).

In summary, an increasing body of literature in recent years has reported that ADSCs may represent perivascular precursor cells located near adipose tissue vessels, contributing to angiogenesis. Sca-1+ ADSCs likely overlap with Sca-1+ VSCs in both function and localization. Moreover, numerous studies have demonstrated the enhanced stemness, proliferative capacity, and adipogenic potential of Sca-1+ ADSCs. Therefore, we used Sca-1 as a marker to purify mouse ADSCs and compared three methods: traditional direct adherence culture, magnetic separation followed by adherence, and adherence followed by magnetic separation which aim to develop a simple and efficient purification method to obtain mouse ADSCs subsets with higher purity, enhanced activity, and more stable characteristics.

## 2 Materials and methods

### 2.1 Animals

Specific pathogen-free (SPF) male C57BL/6J mice, aged 4–6 weeks, were obtained from Hunan Slaike Jingda Laboratory Animal Co., Ltd. The animals were housed under controlled conditions in individually ventilated cages (IVC), with a 12-h light/dark cycle and access to food and water *ad libitum*. The study included three groups (ADSC-A, ADSC-M, and ADSC-AM), with individual mice as experimental units. Randomization was performed and outcome assessors were blinded during data collection and analysis. Sample sizes (*n* = 3 or 5) were determined based on reproducibility and statistical requirements. Mice exhibiting health abnormalities or >10% weight loss during acclimatization were excluded. CO_2_ asphyxiation was used for euthanasia, with gas introduced into a transparent chamber at 20%–30% displacement per minute until respiratory movements ceased. Death was confirmed by the absence of heartbeat and pupil reflex. All procedures adhered to the ARRIVE guidelines for animal research and were approved by the Institutional Animal Care and Use Committee (IACUC) of Chongqing University (Approval number: CQU-IACUC-RE-202410-001).

### 2.2 Isolation of primary mouse ADSCs

C57BL/6J mice (aged 4–6 weeks) were euthanized via carbon dioxide asphyxiation and soaked in 70% ethanol for 3 min. The groin fat was harvested and transferred to a 60-mm culture dish (Labselect, Hefei, China). After rinsing with PBS (Gibco, Grand Island, NY, United States), the tissue was minced into 1–2 mm^3^ fragments and digested in a solution containing 0.25% collagenase type II (Gibco, Grand Island, NY, United States) and 0.1% collagenase type I (Gibco, Grand Island, NY, United States) for 1 h at 37°C. The digestion was terminated with isolation medium, and the mixture was filtered through a 40 μm cell strainer. After centrifugation at 400 g for 10 min, the SVF was resuspended in α-MEM medium with 10% FBS (Gibco, Grand Island, NY, United States), 100 U/mL penicillin, 100 μg/mL streptomycin, and 250 ng/mL amphotericin B (Gibco, Grand Island, NY, United States) for further use.

### 2.3 MACS and adherent culture of mouse ADSCs

#### 2.3.1 ADSC-A

In this method, SVF cells were directly adherent culture. After 48 h of incubation at 37°C in a 5% CO_2_ incubator, non-adherent cells were removed, and medium changes were performed every 2 days. Cells were passaged are 80% confluence using 0.25% trypsin-EDTA solution (Pricella, Wuhan, China). The cells at passage 3 (P3) or passage 4 (P4) were utilized for subsequent experiments.

#### 2.3.2 ADSC-M

In this method, Sca-1+ cells were isolated using magnetic-activated cell sorting (MACS) before adherence culture. Briefly, Sca-1 biotin antibodies (Miltenyi Biotec GmbH, Bergisch Gladbach, Germany) binding to Sca-1 expressed on SVF cells (2.5 × 10^6^ –1 × 10^7^ cells/200 μL), and then were conjugated to anti-biotin microbeads (Miltenyi Biotec GmbH, Bergisch Gladbach, Germany) by incubating at 2°C–8°C for 15 min. The Sca-1+ SVF cells were absorbed onto the MS column (Miltenyi Biotec GmbH, Bergisch Gladbach, Germany) with a maximum cell loading capacity of 2 × 10^8^ cells and a maximum magnetic cell capture capacity of 1 × 10^7^ cells. (Miltenyi Biotec GmbH, Bergisch Gladbach, Germany) under the strong magnetic field, then cultured for further purification and expansion in a 5% CO_2_ incubator at 37°C. Medium changes were performed every 2 days, and cells were passaged at 80% confluence. The cells at passage 3 (P3) or passage 4 (P4) were utilized for subsequent experiments.

#### 2.3.3 ADSC-AM

In this method, adherent cells from the SVF were cultured to passage 3 before undergoing MACS to isolate Sca-1+ ADSCs. Purified cells were cultured with medium changes every 2 days, or passaged at 80% confluence. The cells at passage 3 (P3) or passage 4 (P4) were utilized for subsequent experiments.

### 2.4 Proliferative analysis

#### 2.4.1 Cell counting kit-8 (CCK-8)

ADSC-A, ADSC-M, and ADSC-AM at P3 were seeded in 96-well plates at 2 × 10^3^ cells/well (Labselect, Hefei, China). A total of 100 µL of CCK-8 solution (Beyotime, Shanghai, China) was added to each well, incubated for 1 h, and absorbance was measured at 450 nm on day 1, 2, 3, 4, 5 and 6.

#### 2.4.2 Colony-forming unit fibroblasts (CFU-F)

ADSC-A, ADSC-M, and ADSC-AM at P3 were seeded in six-well plates at 2 × 10^3^ cells/well (Labselect, Hefei, China) and cultured for 14 days. Cells were then fixed with 4% paraformaldehyde (Biosharp, Hefei, China) for 30 min, stained with crystal violet (Beyotime, Shanghai, China) for 20 min, and CFU-Fs were recorded and counted.

### 2.5 Flow cytometry analysis

ADSC-A, ADSC-M, and ADSC-AM at P4 were stained with antibodies targeting CD44-APC (BioLegend, San Diego, CA, United States), CD90-PECD29-PE (BioLegend, San Diego, CA, United States), (BioLegend, San Diego, CA, United States), CD45-PE (BioLegend, San Diego, CA, United States), CD31-PE (BioLegend, San Diego, CA, United States) for identification, targeting Sca-1-PE (BioLegend, San Diego, CA, United States) for comparation the Sca-1+ cell ratios among the three groups. In short, the three groups of cells were stained with five fluorescent antibodies in the dark for 45 min, followed by washing with buffer containing 0.5% BSA (BioLegend, San Diego, CA, United States) and detection by flow cytometry (CytoFLEX).

### 2.6 Induction and differentiation of three lineages

#### 2.6.1 Osteogenic differentiation

In accordance with the manual for the osteogenic induction differentiation kit (Cyagen, Guangzhou, China), ADSC-A, ADSC-M, and ADSC-AM at P3 were seeded in six-well plates coated with 0.1% gelatin at a density of 2 × 10^4^ cells/cm^2^. The osteogenic induction differentiation medium was added to each well in place of the normal medium and changed with fresh medium every 3 days upon reaching 70% confluence. After 3 weeks, cells were fixed with 4% paraformaldehyde and stained with Alizarin Red S (pH 5.1–5.3) for 10 min. The osteogenic differentiation results were observed and recorded under a microscope.

#### 2.6.2 Adipogenic differentiation

In accordance with the manual for the adipogenic induction differentiation kit (Cyagen, Guangzhou, China), ADSC-A, ADSC-M, and ADSC-AM at P3 were seeded in six-well plates coated with 0.1% gelatin at a density of 2 × 10^4^ cells/cm^2^. Adipogenic induction differentiation medium A was added to each well to incubate for 3 days upon reaching 100% confluence, followed by a change to adipogenic differentiation medium B for 1 day. This cycle continued until sufficient lipid droplets appeared, typically within 1–2 weeks. The cells were then fixed with 4% paraformaldehyde and stained with Oil Red O solution (pH 2.1) for 30 min. The adipogenic differentiation results were observed and recorded under a microscope.

#### 2.6.3 Chondrogenic differentiation

In accordance with the manual for the chondrogenic induction differentiation kit (Cyagen, Guangzhou, China), ADSC-A, ADSC-M, and ADSC-AM at P3 were transferred to 15 mL tubes and centrifuged at 250 g for 4 min. The cell pellet was resuspended with chondrogenic premix and centrifuged at 150 g for 5 min. This procedure was repeated twice. The chondrogenic induction differentiation medium was then added to the tubes and changed every 2 days. After 3 weeks of induction, the cartilage spheres were sliced and stained. The chondrogenic differentiation results were observed and recorded under a microscope.

### 2.7 RNA-seq analysis

ADSC-A and ADSC-AM at P3 were lysed using 1 mL of Trizol (Invitrogen, Carlsbad, CA, United States) per 1 × 10^7^ cells to extract RNA in an enzyme-free manner. The total RNA quantity and purity were analyzed using a Bioanalyzer 2100 and an RNA 1000 Nano LabChip Kit (Agilent, CA, United States) with a RIN number >7.0. Poly(A) RNA was purified from the total RNA (5 µg) using poly-T oligo-attached magnetic beads, with two rounds of purification. Following purification, the mRNA fragments were reverse-transcribed to create the final cDNA library according to the mRNA-Seq sample preparation kit protocol (Illumina, San Diego, United States), with an average insert size for the paired-end libraries of 300 bp (±50 bp). Paired-end sequencing was then performed on the Illumina HiSeq 4,000 at LC Science, United States, following the vendor’s recommended protocol.

### 2.8 Bioinformatics analysis of RNA-seq

The raw sequencing data is in FASTQ format. Quality control of the raw sequencing data was performed using fastp (https://github.com/OpenGene/fastp), including adapter removal, deduplication, and filtering of low-quality sequences with default parameters. The sequencing data was aligned to the reference genome (*Homo sapiens*, GRCh38) using HISAT2 (https://ccb.jhu.edu/software/hisat2), generating BAM format files. Transcript assembly and quantification were performed using StringTie (https://ccb.jhu.edu/software/stringtie), with gene expression levels measured in FPKM (fragments per kilobase of exon per million mapped reads; FPKM = total exon fragments/[mapped reads (millions) × exon length (kb)]). Differential gene expression analysis was conducted using the R package edgeR, with genes showing >2-fold or <0.5-fold change and *p*-value <0.05 defined as differentially expressed genes. Finally, functional enrichment analysis of Gene Ontology (GO) terms and KEGG pathways was performed using DAVID (https://david.ncifcrf.gov/).

### 2.9 Statistical analysis

All experiments were repeated at least three to five times. Quantitative data were analyzed using GraphPad Prism 9.0 software (GraphPad Software Inc., San Diego, CA, United States). Tukey’s *post hoc* test was employed for multiple comparisons. CCK-8 assay data were analyzed by two-way ANOVA. Other data were analyzed by one-way ANOVA. Data were expressed as the mean ± SEM with *P* < 0.05 considered statistically significant.

## 3 Results

### 3.1 ADSC-AM displays homogeneous and typical MSC morphology

**FIGURE 1:**
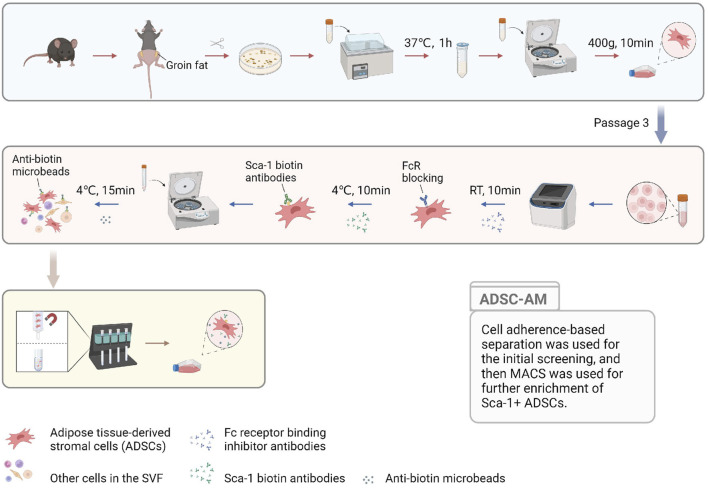
The pattern diagram displaying the ADSC-AM procedure. After isolating the SVF, the cells were cultured to MSCs to gain a growth advantage through media exchange and passaging. Sca-1 positive selection was then performed on third-generation ADSCs using biotin-conjugated Sca-1 antibodies and anti-biotin magnetic beads under a magnetic field (Created with BioRender.com).

To visually describe the three methods for isolating and purifying mouse ADSCs, we compiled the acquisition process into a single figure (Figure 1) to illustrate the sequence, convergence, and differences among the methods. All three methods begin with the acquisition of SVF. Compared to ADSC-A, both ADSC-M and ADSC-AM incorporate magnetic bead sorting, but in different sequences: ADSC-M performs magnetic sorting before adherent culture, while ADSC-AM conducts it afterward. ADSC-A relies on continuous culture for at least three passages to ensure purity. Similarly, ADSC-M requires Sca-1 positive selection followed by adherent purification until the third passage. ADSC-AM, after adherent culture, cells at passage 3 to Sca-1 positive selection. Hence, the culture periods among the three methods showed no significant differences.

**FIGURE 2:**
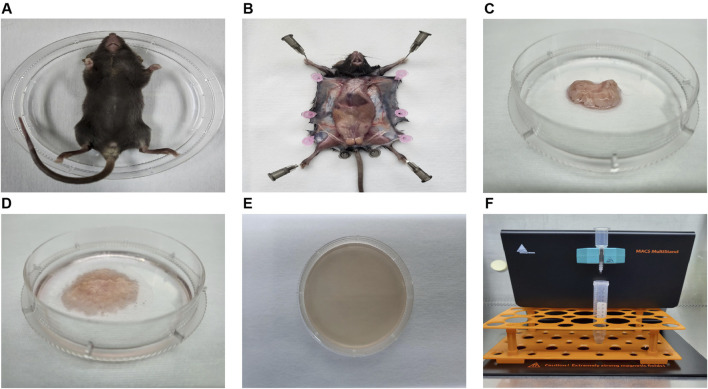
The process diagram illustrating the isolation of mouse ADSCs. **(A)** Mice were sacrificed via CO_2_ asphyxiation and placed in 100-mm sterile Petri dishes. **(B)** The skin was dissected to expose and isolate the inguinal fat pad. **(C)** The inguinal fat pad was collected and transferred to a 60-mm sterile Petri dish. **(D)** Impurities, including lymph nodes, fascia, and blood, were meticulously removed. The adipose tissue was then washed at least three times and finely minced with sterile microscissors. **(E)** Collagenase solution was added to the dish for enzymatic digestion. After digestion, the tissue was gently pipetted until fully liquefied. **(F)** The resulting SVF cells were incubated with magnetic beads and antibodies, followed by Sca-1 positive selection using a magnetic cell sorter.

**FIGURE 3:**
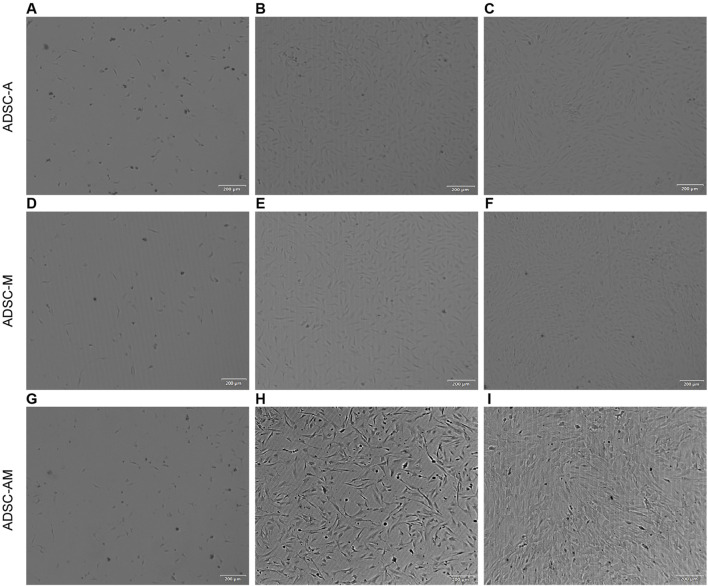
Morphology of mouse ADSCs obtained by three purification methods. **(A, G)** Day 2 (ADSC-A, ADSC-AM): Lipid droplets, tissue debris, and suspended cells are visible. **(B)** Day 4 (ADSC-A): Cells exhibit a more uniform spindle shape (*n* = 3). **(C)** Day 6 (ADSC-A): Cells reach 70%–80% confluence with a whorl-like pattern; some lipid debris and heterogeneous cells remain (*n* = 3). **(D)** Day 2 (ADSC-M): Lower cell density is observed compared to ADSC-A; lipid droplets, tissue fragments, and suspended cells are present (*n* = 3). **(E)** Day 4 (ADSC-M): Spindle morphology becomes more uniform (*n* = 3). **(F)** Day 6 (ADSC-M): Cells reach 70%–80% confluence, with minimal lipid debris and non-uniform cells remaining (*n* = 3). **(H)** Day 2 (ADSC-AM post-MACS): Cells appear elongated, three-dimensional, with a clean background (*n* = 3). **(I)** Day 4 (ADSC-AM post-MACS): Cells reach 80%–90% confluence with a vortex-like arrangement (*n* = 3). Scale bar = 200 µm.

Next, after obtaining the mouse ADSCs according to the procedure (Figure 2), the microscopic morphology of mouse ADSCs derived from the three methods was observed and recorded (Figure 3). By the second day of the first medium exchange in ADSC-A and ADSC-AM, numerous lipid droplets, adipose tissue fragments, and suspended cells were still present, including hematopoietic cells, red blood cells, adipocytes, and irregularly shaped adherent cells (Figures 3A,G). ADSC-M, in contrast, exhibited a significantly reduced number of cells by day 2, indicating effective removal of Sca-1 negative cells (Figure 3D). By day 4, ADSC-A and ADSC-M displayed spindle-shaped characteristics, and ADSC-M showed fewer lipid debris and a more uniform morphology (Figures 3B,E). On day 6, both groups reached 70%–80% confluence, with a distinctive whorl-like arrangement of spindle cells, but ADSC-M showed higher density (Figures 3C,F). ADSC-AM, following Sca-1 sorting after passage 3, displayed a more three-dimensional spindle shape by day 2, with uniform morphology and no visible fat debris (Figures 3H,I). By day 4, ADSC-AM reached 80%–90% confluence and arranged in a whorl-like pattern.

### 3.2 ADSC-AM yields moderate cell numbers with the highest purity

To compare the cell yields obtained by the three purification methods, we used the same weight of adipose tissue from the bilateral inguinal fat pad of a 4-week-old mouse and recorded the cell counts for each passage (Supplementary Table S1). The results indicated that ADSC-A consistently produced the highest number of cells in most passages. However, the number of cells in ADSC-M dropped by over 50% after Sca-1 positive selection, resulting in the lowest overall yield. ADSC-AM showed a cell count similar to ADSC-A from passages 3, although it decreased by 30% after Sca-1 selection—slightly higher than ADSC-M but significantly higher in the subsequent passage. Altogether, the results reveal the following points (Zuk et al., 2001): ADSC-A yields the highest number of cells but likely at lower purity (Gimble et al., 2011); ADSC-AM demonstrates a greater cell yield compared to ADSC-M, which may be related to the initial removal of some competitive hybrid cells by adherent culture in the early stage leading to the growth advantage of mouse ADSCs.

### 3.3 ADSC-AM exhibits enhanced proliferative activity

**FIGURE 4:**
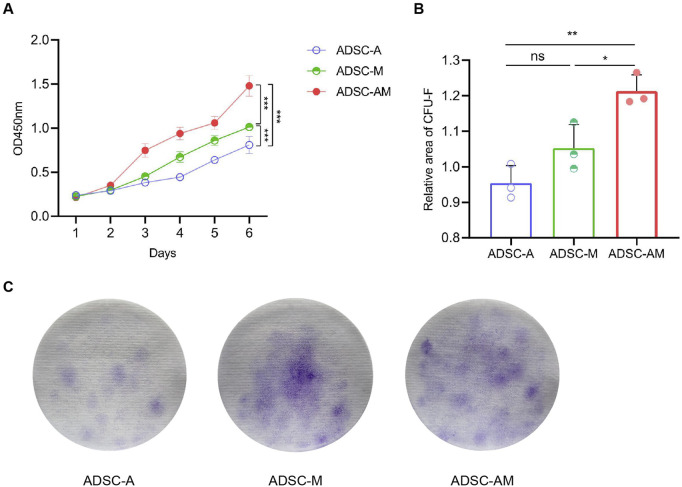
Proliferation of mouse ADSCs obtained by three purification methods. **(A)** Growth curves were measured by a CCK-8 assay (*n* = 5). Data are analyzed by two-way ANOVA and expressed as the mean ± SEM. ns, not significant; *P < 0.05, **P < 0.01, ***P < 0.001. **(B, C)** Colonies were stained with crystal violet **(C)** and CFU-F was counted **(B)** (*n* = 3). Data are analyzed by one-way ANOVA and expressed as the mean ± SEM. ns, not significant; *P < 0.05, **P < 0.01, ***P < 0.001.

To evaluate the proliferative activity of mouse ADSCs obtained by the three methods, we employed the cell counting kit-8 (CCK-8) assay to assess the proliferation of the third generation of ADSC-A, ADSC-M, and ADSC-AM over 6 days (Figure 4A). The results indicated that ADSC-AM appeared to grow faster than ADSC-A and ADSC-M. ADSC-A exhibited the slowest growth, suggesting significant cellular aging. Additionally, we measured the proliferative activity of the third generation of ADSC-A, ADSC-M, and ADSC-AM using the colony-forming unit fibroblast (CFU-F) assay after 14 days (Figures 4B,C), which corroborated the CCK-8 results. After 14 days of inoculation and culture at the same density, ADSC-AM exhibited significantly higher proliferation activity compared to the other two groups, with the highest colony density and number. Therefore, the results demonstrate that ADSC-AM possess stronger proliferative activity than both ADSC-A and ADSC-M.

### 3.4 ADSC-AM displays a purer phenotype and higher Sca-1 positive rate

**FIGURE 5:**
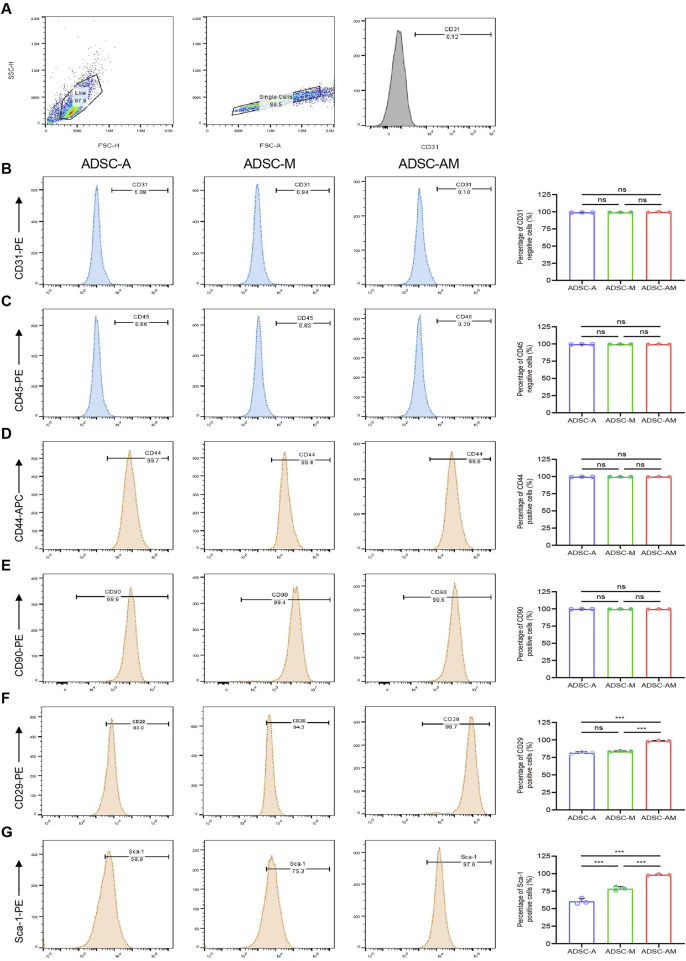
Surface antigen markers of mouse ADSCs obtained by three purification methods. **(A)** Logical gating strategy for all flow cytometry metrics (using CD31 as an example). **(B–G)** Expression levels of the negative cell markers CD31 **(B)** and CD45 **(C)**, as well as the positive expression cell markers CD44 **(D)**, CD90 **(E)**, CD29 **(F)** and Sca-1 **(G)** in each group were detected (*n* = 3). Data were analyzed by one-way ANOVA and expressed as mean ± SEM. ns, not significant; *P < 0.05, **P < 0.01, ***P < 0.001.

To identify and compare the phenotypes of mouse ADSCs obtained through the three methods, we analyzed the fourth passage of mouse ADSCs using flow cytometry. We selected commonly used positive markers for mouse ADSCs: CD44, CD90 and CD29, as well as the endothelial cell marker CD31 and the hematopoietic stem cell marker CD45 establish gating logic (Figure 5A). The flow cytometry results showed that the positive rates of CD44 and CD90 exceeded 90% across all three purification methods (Figures 5D,E). The positive rates of CD29 in both ADSC-A and ADSC-M were above 80% (81.40% ± 0.95% and 83.41% ± 0.54%, respectively), while ADSC-AM showed positive rates exceeding 90% (98.43% ± 0.48%) (Figure 5F). Furthermore, all of the positive rates of CD31 and CD45 were below 5% (Figures 5B,C). To further evaluate the Sca-1 positive rate in mouse ADSCs obtained by the three methods, cells were labeled with a Sca-1 antibody after culturing to the fourth passage. The results revealed that ADSC-A had the lowest Sca-1 positive rate (60.47% ± 2.41%), while the positive rate in ADSC-M increased, though it remained below 80% (78.37% ± 1.76%). In contrast, ADSC-AM exhibited the highest positive rate, exceeding 95% (98.37% ± 0.46%) (Figure 5G).

### 3.5 ADSC-AM shows stronger adipogenic differentiation potential

**FIGURE 6:**
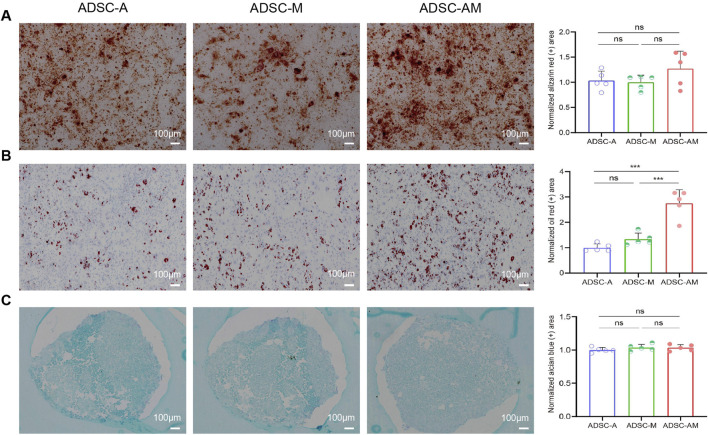
Differentiation potential of mouse ADSCs obtained by three purification methods. **(A)** After osteogenic induction culture, the cells were stained with alizarin red S, and the histogram showed the area of calcium nodules for quantitative analysis (*n* = 5). **(B)** Oil red O staining after adipogenic induction cultures and histogram showing lipid droplet regions for quantitative analysis (*n* = 5). **(C)** After chondrogenic induction culture, alcian blue staining and histograms showing quantitative sorting of mucopolysaccharide regions (*n* = 5). Data were analyzed by one-way ANOVA and expressed as mean ± SEM. ns, not significant; *P < 0.05, **P < 0.01, ***P < 0.001. Scale bar = 100 µm.

To assess the trilineage differentiation potential of the third-passage mouse ADSCs, we performed osteogenic, adipogenic, and chondrogenic differentiation assays. All groups demonstrated successful osteogenesis (Alizarin red staining), adipogenesis (Oil Red O staining), and chondrogenesis (Alcian blue staining) (Figures 6A–C). Notably, while there was no significant difference in the positive areas for calcium nodules and mucopolysaccharides among the three groups, ADSC-AM exhibited a larger positive area for lipid droplets during adipogenic differentiation compared to both ADSC-A and ADSC-M. In short, while all three mouse ADSC types can differentiate into osteoblasts, adipocytes, and chondrocytes, ADSC-AM show superior adipogenic differentiation ability.

### 3.6 Comparison of three methods for mouse ADSCs purification

We summarize the advantages and disadvantages of each purification method (Table 1). ADSC-A yielded the highest cell count but with lower purity. ADSC-M improved purity, but it also resulted in a substantial reduction in cell yield. ADSC-AM offered a balance, achieving medium cell yield with the highest purity and Sca-1+ cell percentage. Both ADSC-A and ADSC-AM are susceptible to contamination during early culture due to cell debris and lipid droplets. Proliferation assays (CCK-8 and CFU-F) showed that ADSC-AM grew more rapidly than ADSC-A and ADSC-M. Conversely, ADSC-AM had the highest proliferative capacity. Altogether, ADSC-AM stands out for its better adipogenic differentiation potential and overall balance between yield and purity.

**TABLE 1:** Comparison of mouse ADSCs characteristics and functions using three purification methods.

Groups	ADSC-A	ADSC-M	ADSC-AM
Cell morphology	Short fusiform	Short fusiform	Elongation
Cell output^a^	High	Low	Middle
Cell purity	Low	Middle	High
Sca-1 positive rate	Low	Middle	High
Contamination risk	High	Middle	High
Senescence risk	High	Middle	Middle
Differentiative capacity	Yes	Yes	Yes
Multiplication capacity	**+ +**	**+ + +**	**+ + + +**
Adpogenic capacity	**+ +**	**+ + +**	**+ + + +**

^a^ The cell yields of fourth-passage cells obtained from the three methods were compared.

### 3.7 Gene expression and pathway enrichment differences between ADSC-AM and ADSC-A

**FIGURE 7:**
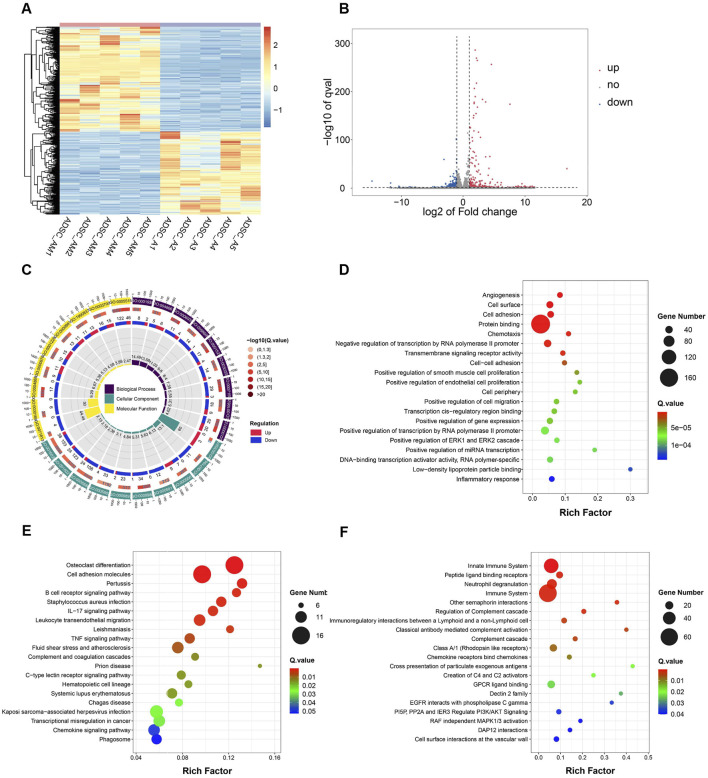
Differential gene expression, function and pathway analysis of ADSC-AM compared with ADSC-A. **(A)** Heatmap of gene expression reveals significant differences between the ADSC-A and ADC-AM groups in the gene clusters and expression patterns (*n* = 5). **(B)** Volcano plot of differential gene expression between the ADSC-A and ADSC-AM groups (*n* = 5). **(C, D)** GO enrichment analysis illustrating the functional categories and the number of differentially expressed genes between the ADSC-A and ADSC-AM groups in biological processes, cellular components and molecular functions (*n* = 5). **(E)** KEGG pathway analysis identifying the enriched signaling pathways and the number of differentially expressed genes between the ADSC-A and ADSC-AM groups (*n* = 5). **(F)** Reactome pathway analysis highlighting the enriched signaling pathways and the number of differentially expressed genes between the ADSC-A and ADSC-AM groups (*n* = 5).

To further investigate transcriptomic differences, paired-end sequencing was performed to compare differential gene expression between ADSC-AM and ADSC-A. Clustering analysis revealed significant differences in gene expression, as depicted in the heat map (Figure 7A). The volcano plot indicated that, compared to ADSC-A, 378 genes were upregulated and 300 genes were downregulated in ADSC-AM (Figure 7B). Subsequently, we focused our analysis on functional and pathway differences between ADSC-AM and ADSC-A. Gene Ontology (GO) enrichment analysis of differentially expressed genes revealed significant enrichment in functions associated with biological processes, cellular components, molecular functions (Figure 7C). The Scatter plot of GO analysis indicated that angiogenesis was the most significantly enriched biological process, followed by cell surface markers in the category of cellular components, and cell adhesion in biological processes. Protein binding emerged as the most enriched molecular function (Figure 7D). These findings suggest that Sca-1 positive selection may influence mouse ADSC surface markers, enhancing their roles in angiogenesis, cell adhesion, and protein binding, supporting the hypothesis of a functional overlap between Sca-1+ ADSCs and Sca-1+ peripheral vascular stem cells. Kyoto Encyclopedia of Genes and Genomes (KEGG) pathway analysis revealed significant enrichment in immune-related pathways, such as B cell receptor signaling, IL-17 signaling, leukocyte transendothelial migration and TNF signaling pathway (Figure 7E). Similarly, Reactome pathway analysis identified enrichment in immune system pathways, including innate immune system, neutrophil degranulation, immunoregulatory interactions between lymphoid and a non-lymphoid cell, activation and regulation of complement cascade. Additionally, Reactome analysis revealed cell surface interactions at the vascular wall and other pathways influencing cellular physiological processes including proliferation, differentiation, survival, apoptosis, and metabolism (Figure 7F).

## 4 Discussion

In recent years, MSCs have attracted considerable attention in regenerative medicine, immunotherapy, and tumor therapy, due to their multidirectional differentiation potential, paracrine functions, immunomodulatory effects, hematopoietic support, and homing abilities (Al Madhoun et al., 2024; Zhao and Liu, 2016; Taheri et al., 2024; Margiana et al., 2022; Huang et al., 2022). Among these, ADSCs are particularly promising for large-scale MSC-based therapeutic production due to their accessibility and potential (Bacakova et al., 2018; Al-Ghadban and Bunnell, 2020; Si et al., 2019). However, despite the rapid rise in clinical trials, the translation of MSCs, including ADSCs, into clinical practice remains hindered by several challenges (Galipeau and Sensébé, 2018; Zhou et al., 2021; Shan et al., 2024). Chief among these is the issue of cellular heterogeneity, which can vary according to species, tissue source, isolation methods, cell passage, and *in vitro* expansion techniques. Even MSCs from the same tissue source can exhibit distinct subpopulations (Mastrolia et al., 2019; Zhang et al., 2022; Liu et al., 2019). Recent studies have shown a close relationship between ADSCs and blood vessels in adipose tissue. On the one hand, a CD201^high^ subpopulation of ADSCs has been identified in the perivascular area of human subcutaneous adipose tissue. This subset regulates DACT2 through the Wnt signaling pathway, affecting the perivascular stem cell phenotype and supporting the view that the adventitia is the native niche of ADSCs (Wang et al., 2024). Additional evidence suggests that ADSCs are integral to the vascular wall, contributing both to adipocyte generation and angiogenesis at the interface between the endothelium and adipocytes (Traktuev et al., 2008). On the other hand, researchers increasingly propose that perivascular ADSCs represent vascular precursor cells at various stages of differentiation (Lin et al., 2008; Lin et al., 2010). Firstly, a substantial body of evidence from various studies suggests that perivascular cells may serve as potential ADSCs. They share multiple characteristics, similar gene profiles, similar antigen markers, etc. (Traktuev et al., 2008; Geevarghese and Herman, 2014; Wu et al., 2023). Secondly, Perivascular ADSCs can also differentiate into vascular endothelial cells and vascular smooth muscle cells (Forghani et al., 2020; Shang et al., 2019; Yogi et al., 2021). For instance, Xie et al. used single-cell sequencing to identify the CELEC11A + PV- perivascular subset of ADSCs as a source for vascular smooth muscle differentiation (Xie et al., 2022). These findings suggest that in adipose tissue, ADSCs are localized in the perivascular region and serve as a reservoir of cells that can differentiate into perivascular cells, explaining why transplantation of the SVF—rather than transplantation of mature adipocytes—improves angiogenesis and blood supply in ischemic tissue (Zhang et al., 2021; Sumi et al., 2007).

Accumulating evidence suggests that ADSCs may function as VSCs, exhibiting different stages and differentiation pathways influenced by their dynamic growth capacity and pluripotency characteristics within the vascular system. Research have been reported that ADSCs labeled with CD34+ CD31− CD104B-SMA-coexist with pericytes and endothelial cells in capillaries, and show as specialized fibroblasts in the adventitia of larger vessels (Zimmerlin et al., 2013). As early as 2007, Yamamoto et al. successfully detected Sca-1 expression in mouse adipose tissue using immunohistochemical staining (Yamamoto et al., 2007). Subsequently, in 2008, Rodenheffer et al. identified Sca-1 as a key marker for screening adipose stem cells (Rodeheffer et al., 2008). With widely recognized as a marker for enriching stem cells across various tissues, An increasing number of studies have utilized Sca-1 to label subsets of VSCs, which demonstrate enhanced proliferative capacity and vascular repair ability (Tang et al., 2020). While there is no consensus that ADSCs are equivalent to VSCs, numerous studies have indicated significant functional and localization overlaps between Sca-1+ VSCs and Sca-1+ ADSCs (Mižíková et al., 2022; Lin et al., 2008; Lin et al., 2010; James et al., 2012). Additionally, subsequent research has confirmed that Sca-1+ MSCs possess distinct advantages, including enhanced stemness, increased proliferation activity, and superior trilineage differentiation potential (Steenhuis et al., 2008).

While Sca-1 is not a specific marker for mouse ADSCs and can also be expressed on the surface of endothelial and hematopoietic stem cells within the SVF cell population, our study found that the direct adherence method employed in all three purification techniques effectively removed most endothelial and hematopoietic cells, as demonstrated by flow cytometry identification results. It is noteworthy that flow cytometry results demonstrated consistently high expression (>90%) of CD44 and CD90 across all three groups without significant differences. This observation aligns with the established understanding that CD44 and CD90 are stable and ubiquitously expressed surface markers of mouse ADSCs. While Sca-1 sorting effectively enhances the purity and function of ADSCs, it does not necessarily impact the expression of pan-ADSC markers like CD44 and CD90, which are shared across heterogeneous subpopulations. In contrast, CD29 and Sca-1 appear to be more sensitive indicators of functional enhancement and purification efficiency in our model, supporting their utility in characterizing high-quality ADSC subsets. Furthermore, our comparison of cell yields and proliferation activity among the three methods revealed a correlation between the Sca-1 positive rate and enhanced cell activity and proliferation capacity. Although some mouse ADSCs that do not express Sca-1 are lost during Sca-1 magnetic bead sorting, resulting in lower cell yields for ADSC-M and ADSC-AM compared to ADSC-A, this trade-off is justified by the higher purity obtained. Additionally, we noted that the proliferative capacity of ADSC-A diminished with increasing passages, accompanied by signs of cellular aging. In contrast, ADSC-AM, with their higher Sca-1 positive rate, demonstrated significantly enhanced proliferative activity, allowing them to surpass ADSC-M in cell numbers. Furthermore, our investigation into the differentiation potential of the three lineages also showed that while all cell types could be successfully induced, ADSC-AM exhibited a notably stronger adipogenic differentiation potential. Lastly, Angiogenesis-related processes were significantly enriched in GO analysis, suggesting that ADSC-AM cells possess stronger pro-angiogenic potential. Other related pathways, such as leukocyte transendothelial migration enriched in KEGG analysis and vascular wall interaction enriched in Reactome analysis support a vascular-associated functional enhancement. Immune-related pathways, such as IL-17, TNF signaling, and complement activation, suggest a stronger immunomodulatory capacity in ADSC-AM, which supports their potential application in inflammation-related regenerative therapies. Cell adhesion and protein binding functions may enhance ADSC-AM homing ability, survival, and integration in tissue environments, consistent with their increased proliferative and differentiation activity observed *in vitro*. These transcriptomic features correlate well with our observed biological outcomes, such as increased proliferative capacity and adipogenic differentiation, and provide functional explanations for the superiority of the ADSC-AM subset.

The observed results can be attributed to several factors. First, ADSC-A did not undergo magnetic bead sorting, which allowed for the retention of a greater number of Sca-1- cells—potentially including some ADSCs—that possess certain adhesion abilities for division and expansion during each digestion passage. Second, the discrepancy in cell yield between ADSC-M and ADSC-AM may stem from the fact that the expansion of ADSCs is highly dependent on cell density, particularly during the early stages of adherent culture. Many Sca-1- ADSC-M were lost due to the positive selection of Sca-1 before the ADSC-M could expand to sufficient numbers. In contrast, adherence to the third generation before magnetic cell sorting, that is, ADSC-AM leveraged their relatively stronger adhesion capabilities and adaptability to the *in vitro* environment, enabling them to achieve higher yields and a distinct proliferative advantage. By this point, most heterogeneous cells that do not adhere well or have poor adhesion abilities, as well as other weaker cells that cannot compete with ADSC-AM, were effectively screened out. Overall, ADSC-AM undergoes adherent culture followed by Sca-1− positive selection, which improves ADSC purity and enriches Sca-1+ ADSCs to obtain a functionally enhanced subpopulation. In contrast to ADSC-M, which differs in procedural sequence, ADSC-AM gained a proliferative advantage and formed the dominant cell population before Sca-1 positive selection, achieved through early adherent culture and serial passaging to further eliminate other subgroups of cells competing for living space. This strategy first selects ADSC populations with greater proliferative activity, then further isolates Sca-1+ ADSC subpopulations with higher purity and superior functional properties. However, ADSC-M involves immediate magnetic sorting after SVF isolation. Premature Sca-1 selection may compromise the proliferative advantage of ADSCs, and prolonged manual operations could cause irreversible damage to the cells. These conclusions are corroborated by the comparative evaluation of biological functional characteristics between ADSC-M and ADSC-AM (Table 1).

In this study, we developed a novel procedure for the isolation and purification of Sca-1-positive mouse ADSCs, referred to as the ADSC-AM method. Compared to traditional direct adherence (ADSC-A) and magnetic sorting methods (ADSC-M), the ADSC-AM approach achieved higher cell purity and an increased Sca-1 positive rate. Although the surface markers of mouse ADSCs are not yet fully characterized, our findings indicated that ADSC-AM with three high-expressed primary positive indicators and two low-expressed primary negative indicators, not only met the ISCT basic standards but also exhibited enhanced proliferative capacity, colony-forming ability, and multilineage differentiation potential, particularly in adipogenesis. These results underscore the potential of ADSC-AM in tissue engineering and regenerative medicine. Furthermore, transcriptomic analysis revealed specific gene expression profiles associated with angiogenesis and immune regulation, supporting the functional promise of these cells in tissue repair and immunotherapy. However, several limitations of this study should be acknowledged. First, while Sca-1 serves as an effective marker for ADSC enrichment, it is not specific to ADSCs and is expressed on other cell types within the SVF, which may allow non-target cells to persist in the purified population. Second, this study did not investigate important functional characteristics of ADSC-AM, such as migration and anti-apoptotic effects, nor did it include functional validation experiments to confirm its enhanced angiogenic and immunomodulatory capabilities. We plan to further explore these aspects in future research. Third, in our study, ADSC-AM was cultured up to passage 10, and the cells maintained normal basic morphology and functional properties. However, the impact of more prolonged *in vitro* culture on ADSCs included chromosomal stability has not yet been assessed, which is critical for long-term safety and clinical applicability of stem cell-based therapies. Additionally, the optimization of MSC culture conditions (such as serum-free culture systems) remains an area of active exploration, as these factors are crucial for mitigating potential adverse effects during long-term ADSC expansion. Future studies are warranted to investigate these aspects comprehensively. Lastly, all experiments were conducted in mouse ADSCs rather than human ADSCs, as our primary focus was on ensuring the uniformity of preclinical research models. However, the success of the ADSC-AM method in murine models provides a transferable technical framework for human ADSC purification, though its translation requires addressing species-specific marker differences and optimizing sorting strategies. If successfully adapted, this approach holds promise for delivering high-purity, functionally enhanced ADSC subpopulations to human regenerative medicine, advancing their clinical applications in refractory diseases such as tissue ischemia and immune disorders. The ADSC-AM procedure to human ADSCs, as well as characterizing the properties and functions of isolated human ADSCs is quite necessary and remains a highly valuable research direction for our future studies.

## 5 Conclusion

In conclusion, our study identified Sca-1 as an effective marker for the purification of mouse ADSCs and finally developed a simple yet highly efficient procedure (ADSC-AM) for purifying and enriching Sca-1-positive mouse ADSCs. These cells exhibited superior proliferative capacity, colony-forming ability, and adipogenic potential. Accordingly, our findings shed light on the heterogeneity of mouse ADSCs, which has previously complicated preclinical research, while offering a simple and efficient method for mouse ADSC separation and purification, promising valuable insights for future ADSC research and applications.

## Data Availability

The data presented in the study are deposited in the https://www.ncbi.nlm.nih.gov/sra, accession number PRJNA1257523.
