# Supplementary material for: Optimal Sca-1-based procedure for purifying mouse adipose-derived mesenchymal stem cells with enhanced proliferative and differentiation potential
Source: Front Cell Dev Biol. 2025 May 16;13:1566670. doi: 10.3389/fcell.2025.1566670 (PMC12122437; doi:10.3389/fcell.2025.1566670)
Supplement: Supplementary file 1 [file Table1.docx]

| **Supplementary Table S1.** Comparison of mouse ADSCs yields using three purification methods. | | | | | | | |
| --- | --- | --- | --- | --- | --- | --- | --- |
| Passage number | ADSC-A Cells (×10^6^) | ADSC-M Cells (×10^6^) | | | ADSC-AM Cells (×10^6^) | | |
|  | Adherence | Pre-MACS | Post-MACS | Adherence | Adherence | Pre-MACS | Post-MACS |
| Passage 0 | 14.43 ± 0.62 | 14.87 ± 0.17 | 6.52 ± 0.13 |  | 14.77 ± 0.31 |  |  |
| Passage 1^a^ | 2.26 ± 0.11 |  |  | 2.03 ± 0.10 | 2.30 ± 0.13 |  |  |
| Passage 2 | 5.47 ± 0.14 |  |  | 4.52 ± 0.23 | 5.64 ± 0.12 |  |  |
| Passage 3 | 17.40 ± 0.15 |  |  | 12.07 ± 0.29 |  | 18.65 ± 0.07 | 13.20 ± 0.27 |
| Passage 4 | 28.13 ± 0.23 |  |  | 19.72 ± 0.54 | 25.21 ± 0.14 |  |  |
| a. The cell numbers of first-passage cells obtained from the three methods were similar. | | | | | | | |
